# The epigenetic legacy of illicit drugs: developmental exposures and late-life phenotypes

**DOI:** 10.1093/eep/dvz022

**Published:** 2019-11-13

**Authors:** Nicole M Wanner, Mathia L Colwell, Christopher Faulk

**Affiliations:** 1 Department of Veterinary and Biomedical Sciences, University of Minnesota College of Veterinary Medicine, 1988 Fitch Ave, 495B AnSc/VetMed, St. Paul, MN 55108, USA; 2 Department of Animal Science, University of Minnesota College of Food, Agricultural and Natural Resource Natural Resource Sciences, 1988 Fitch Ave, 495B AnSc/VetMed, St. Paul, MN 55108, USA

**Keywords:** developmental programing, epigenetics, illicit drugs, addiction, opioids, cannabinoids

## Abstract

The effects of *in utero* exposure to illicit drugs on adult offspring are a significant and widespread but understudied global health concern, particularly in light of the growing opioid epidemic and emerging therapeutic uses for cannabis, ketamine, and MDMA. Epigenetic mechanisms including DNA methylation, histone modifications, and expression of non-coding RNAs provide a mechanistic link between the prenatal environment and health consequences years beyond the original exposure, and shifts in the epigenome present in early life or adolescence can lead to disease states only appearing during adulthood. The current review summarizes the literature assessing effects of perinatal illicit drug exposure on adult disease phenotypes as mediated by perturbations of the epigenome. Both behavioral and somatic phenotypes are included and studies reporting clinical data in adult offspring, epigenetic readouts in offspring of any age, or both phenotypic and epigenetic measures are prioritized. Studies of licit substances of abuse (i.e. alcohol, nicotine) are excluded with a focus on cannabis, psychostimulants, opioids, and psychedelics; current issues in the field and areas of interest for further investigation are also discussed.

## Introduction

Maternal use of illicit drugs represents a common, concentrated, and repeated *in utero* exposure with established detrimental consequences for offspring in the neonatal and childhood periods, however, it is often less clear how these exposures impact perinatally exposed adults. The developmental origins of health and disease paradigm states that early life events play a critical role in shaping adult health and disease phenotypes [1]. Indeed, environmental factors such as diet, lifestyle, adverse events, and toxicant exposures have since become part of the etiology for a variety of complex conditions including cancer, neurological disorders, and aging [2]. Epigenetics is a mechanism through which the environment can interact with the genome to generate plastic phenotypic responses and includes DNA methylation, histone modifications, and non-coding RNAs. The most well-studied epigenetic mark, DNA methylation, occurs at CpG dinucleotides and acts to control gene regulation and transcription in a long-term manner [3]. The epigenome is particularly sensitive to dysregulation by toxicants or other stressors during the global resetting and mitotic propagation of epigenetic marks in early development, and the patterns established during these critical periods can persist and lead to disease phenotypes in adulthood [4, 5]. Illicit drugs as an exposure exist in a different category from other stressors. These substances are the most common exogenous chemicals to which humans are exposed, with the exception of those found in food and perhaps caffeine. In contrast to exposure to other toxicants, which is often incidental and at low concentrations, illicit drugs are voluntarily consumed in large, often chronically increasing doses with high frequency over long periods of time. The goal of the current review is to highlight links between perinatal drug exposure, adult-onset disease phenotypes, and epigenetic mechanisms of gene regulation. Where these direct links are lacking, relevant papers from the literature are connected to identify possible mechanisms or phenotypes of interest for future investigation. A primary focus on prenatal cannabinoid exposure is presented with additional discussion of cocaine, opioids, amphetamines, and psychedelics where available; findings from the literature are grouped by phenotype rather than by drug to highlight instances of shared downstream pathophysiology between substances with differing mechanisms of action.

## Behavioral Phenotypes

### Addiction Vulnerability

A key phenotype of interest for *in utero* illicit drug exposure is the vulnerability to future substance abuse in adulthood. Substance use disorder (SUD), which is defined by the inability to cease drug use despite negative consequences, is a complex and debilitating disease with a network of genetic, epigenetic, and environmental risk factors affecting ∼71 million people each year [6–15]. Changes in the mesolimbic pathway are frequently evaluated in models of SUD; this dopaminergic tract connects the ventral tegmental area (VTA) in the midbrain to the nucleus accumbens (NAc) in the ventral striatum of the basal ganglia and is associated with reward behavior and habitual learning [16, 17]. Daily exposure to methamphetamine (METH) throughout gestation in rodents leads to strong increases in levels of dopamine and its metabolites in the NAc in addition to increased behavioral responsiveness to an acute dose of the same drug in adult offspring [18]. Prenatal METH may also predispose offspring to use of other psychostimulants via epigenetic modulation, with work by Itzhak *et al*. showing that increased behavioral sensitivity to cocaine following prenatal METH exposure was associated with widespread methylation changes at intergenic CpG islands and gene promoters in the hippocampus of F1 animals [19]. Although more targeted analyses will be required to uncover the mechanistic relationship between methylation changes and intergenerational behavioral outcomes, differentially methylated regions (DMRs) were found in promoters of genes with more direct evidence of influence on addiction and reward in this study. Chemical blockade of Gsk-3-β, for example, is associated with reduced ketamine self-administration in rats [20]. Global changes in DNA methylation have also been observed with prenatal exposure to other psychostimulants, but the persistence of the exposure’s effects can be more ambiguous. A study by Novikova *et al*. found over 400 differentially methylated CpG islands and corresponding gene expression changes in neonatal mice prenatally exposed to cocaine, however, adolescent mice showed distinct dysregulated methylation and expression profiles from their younger counterparts [21]. With no behavioral outcomes it is impossible to extrapolate the biological consequences of these differences, however, the plastic nature of epigenetic changes across time indicates a need for attention to longitudinal changes throughout the life course as a response to developmental exposure (Table 1).

**Table 1:** selected animal models of perinatal drug exposure with epigenetic and/or adult phenotypic endpoints

Model	Phenotype	Drug	Exposure paradigm	Offspring characteristics	Behavioral assay (if applicable)	Molecular target	Key results
Rat	Addiction vulnerability—dopaminergic signaling	Cannabis (WIN)	Daily increasing dose (1, 2, 4 mg/kg) SQ q12h for 3 days during maternal adolescence (non-gestational)	Adult females	Morphine-induced locomotor sensitization	mRNA level	Increased behavioral response to morphine, increased OPRM1 mRNA; normal Drd1, Drd2, cFos, FosB expression in NAc [25]
Rat	Addiction vulnerability—dopaminergic signaling	Cannabis (THC)	0.15 mg/kg IV daily from GD5-PND2, offspring fostered by control dams thereafter	Neonatal (PND2) and adult males	Morphine place conditioning	Histone lysine methylation	Reduced NAc Drd2 mRNA at PND2 and in adulthood, increased Drd2 2meH3K9 (repressive), decreased 3meH3K4 and RNAPII in adults [22]
Rat	Addiction vulnerability—glutamatergic signaling	Cannabis (THC)	Dual parental adolescent exposure of 1.5 mg/kg IP every third day from PND 28–49; offspring fostered by control dams	Adult males and females	NA	Genome-wide DNA methylation	1027 DMRs in introns, exons, and intergenic intervals; depleted in gene promoters. DMRs and expression changes associated with GluR synaptic regulation in NAc [31]
Mouse	Cognitive impairment	Cocaine	20 mg/kg SQ twice daily on GD8–18	Adolescent (PD32–36) and adult (60–65) males and females	Morris water maze, open field test	DNA methylation	Elevation of hippocampal Igf-II DMR 2 methylation, upregulation of l-methionine and DNA methyltransferase 1. Intra-hippocampal injection of recombinant Igf-II reactivated repressed calcium calmodulin kinase II α and reversed cognitive deficits in exposed offspring [121]
Mouse	General/molecular	Cocaine	20 mg/kg SQ on GD8–19	Neonatal (P3) and adolescent (P30) males and females	NA	DNA methylation (CpG islands)	492 differentially methylated CGIs at P3 with loci returning to normal or reversing direction of change and presence of previously unaffected CGIs at P30 in hippocampal pyramidal neurons. Subsequent gene expression changes for genes with modified CGIs in promoter [21]
Rat	Cardiovascular	Cocaine	15 mg/kg IP twice daily from GD15–20	Adult males and females	NA	PKCε) promoter methylation	Hypermethylation of promoter CpGs associated with PKCε mRNA downregulation, loss of cardioprotection in the face of ischemic challenge [95]
Rat	Aging	Cocaine	20 or 40 mg/kg SQ twice daily from GD7–20	Longitudinal measures (neonatal–adulthood) in males and females	NA	Lifespan, aged body weight	10–12% decrease in total lifespan in female offspring and 7–9% decrease in male offspring; reduced weights in old age for both sexes [101]

Epigenetically mediated perturbations specific to dopaminergic signaling pathways have also been identified for individuals prenatally exposed to cannabis (*Cannabis sativa*). The primary psychoactive component of cannabis, Δ-9-tetrahydrocannabinol (THC), interacts with endocannabinoid receptors (CB1) in the brain resulting in its euphoric and relaxing effects. DiNieri *et al*. demonstrated that human infants exposed to cannabis *in utero* exhibit decreased dopamine receptor 2 (DRD2) mRNA in NAc [22]; a parallel rodent model of prenatal THC exposure in the same study revealed matching expression changes accompanied by increased repressive and decreased activating histone marks as well as increased sensitivity to the rewarding effects of morphine in adult offspring. Strong evidence in the literature supports interactions between the endocannabinoid and opioid systems in relation to addictive behaviors, and the long-term preservation of this phenotype is consistent with the role of H3 di- and trimethylation in lifelong tissue-specific gene silencing [23, 24]. In contrast, a study by Vassoler *et al*. investigating the effects of maternal adolescent exposure to the synthetic cannabinoid receptor agonist WIN-55 212 (WIN) on adult offspring addiction behavior found no changes in baseline dopamine receptor expression in the NAc of offspring, however, behavioral sensitization to morphine remained present in the offspring of exposed mothers [25]. Behavioral changes were instead correlated with changes in expression of mu-opioid receptor (OPRM1). OPRM1 is the primary receptor targeted by opioids, including heroin, and activation leads to dopamine release and feelings of euphoria that contribute to establishing and perpetuating addictive behaviors [26–28]. Human epigenetic studies show increased OPRM1 expression in infants exposed to cannabis during pregnancy [29]. Interestingly, DNA methylation of dopamine receptors was not significantly affected by exposure to cannabis in neonates exposed *in utero*, however, the use of multiple illicit and licit substances and a lack of longitudinal information make the adult consequences of exposure difficult to extrapolate. Yet, the presence of a shared phenotypic outcome for adults with two vastly different exposure windows (prenatal versus gametic) provides evidence against the idea that the observed changes in SUD vulnerability are the result of direct drug effects through the placenta, and also emphasizes the extended period of vulnerability of the oocyte and embryo to environmental insults.

The excitatory neurotransmitter glutamate also plays an important role in the neurochemical reward pathways of drug addiction in the VTA and NAc. Glutamatergic signaling is necessary for the development and preservation of addictive behavior and is heavily influenced by dopaminergic input [16, 30]. Direct and indirect effects on glutamatergic signaling were identified in a genome-wide methylation study of adult rats exposed perinatally to THC by Watson *et al*.; over 1000 DMRs were identified in the NAc with DMRs being primarily associated with glutamate (GluR) and kainate receptors, pre- and postsynaptic ion channels, and other genes enriched for protein–protein interactions with glutamatergic synaptic transmission genes [31]. Changes in GluR gene expression associated with cannabinoids have been reported elsewhere but appear to manifest differently in early and late adulthood. A study in rodents by Szutorisz *et al*. found that GluR expression changes associated with THC exposure in rodents were correlated with increased heroin self-administration at all time points, but while adolescent offspring exhibited increased GluR and endocannabinoid receptor expression in NAc adults had normal expression in NAc and decreased expression in the dorsal striatum [32]. The influence of glutamatergic activity in the lateral subregion of the dorsal striatum on habit learning and compulsive behavior has been well documented, and early changes in the NAc followed by modifications in this region have been linked to the transition from reward-based drug use to compulsive abuse seen in the progression of human SUD [32, 33].

While it is difficult to generate definitive conclusions based on the limited number of studies, the available literature indicates that prenatal exposure to a number of illicit drugs can lead to increased vulnerability to addictive behavior in adult offspring and is associated with changes in DNA methylation and histone modifications in key reward pathways of the brain. Expansion of epigenetic analyses to include other regions of the brain involved in addiction may also help to clarify the epigenetic mechanisms of prenatal drug exposure phenotypes; DNA methylation has been found to be required for the formation of stimulus–reward association learning in dopaminergic neurons of the VTA, for example [34]. Further interrogation of changes in the reward pathways of the brain will be required to allow integration of prenatal exposure into the clinical understanding of SUD in the future (Table 2).

**Table 2:** human studies of maternal drug use with epigenetic endpoints

Phenotype	Drug(s)	F1 characteristics	Molecular target	Key results with citation
Addiction vulnerability	Cannabis; tobacco and/or alcohol	Fetal (18–22 weeks gestation), male and female, multiple ethnicities	mRNA level	DRD2 expression in NAc of exposed offspring [22]
Addiction vulnerability	Methadone; tobacco, heroin, cannabis, benzodiazepines	Neonatal (24–72 hours)	DNA methylation	Increased methylation in promoters and/or first exons of OPRM1, ABCB1, and CYP2D6 in buccal swabs of opioid-exposed offspring [29]
Addiction vulnerability	Cannabis; heroin, amphetamines, cocaine	Neonatal (8 weeks)	DNA methylation	No changes in DNA methylation at DRD4 promoter in buccal swabs of exposed offspring [122]

Primary exposures of interest are listed along with secondary background drug exposures reported by the authors.

### Major Psychiatric Disorders

Vulnerability to major psychiatric disorders including schizophrenia, anxiety, and depression are behavioral outcomes of interest for perinatal drug exposure due in part to the established deleterious effects of these substances on neurodevelopment. Prenatal exposure to cocaine, cannabis, METH, ‘ecstasy’, or 3,4-methylenedioxymethamphetamine (MDMA), and other drugs has been shown to lead to dopaminergic and serotonergic gene dysregulation and neurotoxicity, delayed neurodevelopmental milestones, and other changes in neonates and adolescents [35–39]. Neurobehavioral pathways relevant to these disorders overlap considerably with those involved in SUD and comorbidity is alarmingly frequent; 8.5 million adults suffer from both a mental health disorder and SUD in the USA annually [40]. Epigenetic mechanisms are critical to the synthesis of major psychiatric disorder etiopathology and additionally interact with predisposing genetic markers and early life environmental adversity [41]. Dysregulated signaling of brain-derived neurotrophic factor (BDNF), a member of the neurotrophin family of growth factors important for proper neurodevelopment as well as higher cognitive functioning, has been implicated in the etiology of several psychiatric disorders [42–44]. BDNF methylation has been established as a biomarker of early life adversity, particularly bisphenol A (BPA) exposure and maternal maltreatment, in rodents and its expression is decreased in the brains of human suicide victims [45–47]. Downregulation of BDNF has also been associated with increased DNA methyltransferase (DNMT) binding to BDNF promoters in glutamatergic neurons of schizophrenia-affected individuals, highlighting the importance of epigenetic mechanisms in the regulation of this pathway [48]. Mice gestationally exposed to cocaine exhibit decreased BDNF mRNA expression in the frontal cortex at embryonic day 15; interestingly, changes are reversed and sex-specific in adulthood with male offspring showing increased BDNF mRNA and protein levels resulting from histone H3 hyperacetylation in the promoter at postnatal day 60. Adult females showed no expression abnormalities [49, 50]. No differences in expression or histone acetylation were observed at PND16 despite clear early developmental and late-life perturbations, suggesting that the effects of *in utero* drug exposure on adults may be difficult to predict based on changes identified earlier in the life course.

Early life stress and BDNF signaling have also been linked to stress pathways in the brain. BDNF polymorphisms and differential methylation have been shown to modulate stress responses via the hypothalamic–pituitary axis [51, 52]. Intermittent prenatal exposure to MDMA has been associated with increased pro-opiomelanocortin (POMC) and corticosterone levels in the brain of adult offspring; interestingly, no behavioral effects on depression or anxiety behavior were observed [53]. Anxiety behavior measured by elevated plus maze was also not affected by prenatal METH in a study by Schutová *et al*., indicating that developmental exposure to psychostimulants may not result in persistent effects on predisposition for anxiety disorders in adulthood [54]. Dysregulation of the monoamine neurotransmitter serotonin (5-hydroxytryptamine; 5-HT) has also been identified in association with multiple psychiatric disorders, and pharmaceutical modification of 5-HT levels via selective serotonin reuptake inhibitors is a common first-line therapy in clinical depression [55, 56]. Interactions between serotonergic signaling and the endocannabinoid system present a mechanistic link between cannabis exposure and late-life psychiatric phenotypes [57–59]. Prenatal exposure to THC, the psychoactive component of cannabis, has been shown to modify 5-HT levels in multiple brain regions important in mood regulation including the midbrain raphe nuclei, a cluster putatively targeted by some antidepressants [60]. These results are intriguing given the contradictory finding that administration of cannabidiol (CBD), an increasingly popular non-psychoactive phytocannabinoid, can reduce symptoms of depression and anxiety in adults [61–64]. Additional research will be required to delineate the differential consequences of cannabis exposure during development and adulthood as well as the effects of interactions between THC, CBD, and hundreds of terpenes and polyphenols found in cannabis on behavioral phenotypes.

### Memory and Cognitive Function

Perinatal drug exposure, particularly to cannabinoids, has also been associated with defects in memory and cognitive function persisting into adulthood [65–67]. Many discrete regions of the brain are associated with memory formation, with the primary areas being hippocampus (declarative, episodic, spatial, and recognition memory), cerebellum (procedural and motor memory), prefrontal cortex (semantic and working memory), and amygdala (emotional memory) [68–70]. These functional assignments are broad generalizations, however, and each specific type of memory involves elaborate connections to surrounding subregions [71–75]. The effects of THC on glutamate signaling appear to be relevant to memory formation in addition to SUD vulnerability, albeit in a different region of the brain; the cerebellum, which has traditionally been thought to primarily affect locomotion, has recently been shown to play a role in memory and cognition [76–79]. A study by Suárez *et al*. showed that prenatal exposure to THC led to downregulation of glutamate transporter genes GLAST and EAAC1 in the cerebellum in addition to significant cognitive and memory deficits in adult offspring [80]. The synthetic THC analog WIN has also been shown to affect long-term memory retention in perinatally exposed rats in association with changes to long-term potentiation and glutamate release in the hippocampus [81]. In addition to its intergenerational effects on memory THC has also been shown to affect adult cognitive function through changes in glutamate signaling; Campolongo *et al*. found that perinatal THC in rats led to perturbed cortical glutamatergic and noradrenergic gene expression in addition to long-term cognitive deficits [82].

While the majority of studies focus on the effects of cannabinoids (likely due to their increasing use and perceived safety), others have evaluated the effects of perinatal exposure to other illicit drugs on memory and cognition in adult offspring. METH, for example, was found to affect recognition memory but not spatial memory in the adult male offspring of exposed females indicating a possible subtle effect on the hippocampus which is responsible for both memory types [83]. Interestingly, a single acute exposure to METH in adulthood led to worsening of the memory phenotype in perinatally exposed animals but not naive animals, indicating that exposed individuals exposed to METH *in utero* may be more severely affected by amphetamine use in adulthood in addition to showing baseline memory deficits as a consequence of previous exposure. A similar compromised response to stressors in adulthood has been observed in association with maternal diet, with mice exposed to high-fat diet *in utero* exhibiting amplified liver steatosis and diet-induced obesity phenotypes in response to dietary challenge in adulthood [84, 85]. The mechanisms linking epigenetic changes to compromised adult response to re-exposure remain to be elucidated. The finding that amplified obesity phenotypes associated with maternal high-fat diet can be buffered with methyl donors is a promising finding worth evaluating in the context of drug exposure [86, 87], however, particularly given the shared reward circuitry between drugs of abuse and highly palatable foods [88]. Understanding the causes of altered response to drug re-challenge in late life, particularly in cases where a small number of exposures can significantly worsen or unmask the latent phenotype, will be vital for making informed preventative recommendations for individuals with a familial history of substance use in the future.

## Somatic Phenotypes

### Cardiovascular Disease

Cardiovascular disease is the leading cause of death worldwide and appears to be influenced by both heritable and environmental factors. There is strong evidence that prenatal environment can impact cardiovascular disease risk during adulthood [89], and chronic cocaine use in adults can lead to ischemic organ injury and cardiovascular disease [90, 91]. Bae and Zhang showed that prenatal cocaine exposure increased vulnerability to reperfusion injury in male offspring and additionally induced abnormal postnatal cardiomyocyte apoptosis [92]. The deleterious effects of cocaine on the cardiovascular system are most often studied in the context of neurochemical and vasoconstrictive pathways, however, recent work has also emphasized the role of epigenetic mechanisms in fetal programing of this phenotype [93, 94]. A study evaluating the effects of prenatal cocaine exposure on the neonatal rat heart by Zhang *et al*. found that exposure led to decreased mRNA and protein levels of the cardioprotective gene protein kinase Cε (PKCε) associated with hypermethylation of CpG dinucleotides in the gene promoter [95]. Additional work utilizing *ex vivo* cocaine exposure in GD17 fetal rat hearts established a causal relationship between PKCε downregulation and hypermethylation at SP1 transcription factor binding sites in the promoter via chromatin immunoprecipitation and targeted mutation assays combined with the use of DNA methylation inhibitors [96].

It is clear that downregulation of PKCε plays a key role in cardioprotection during both ischemia and reperfusion injury in the adult heart, however, there is evidence that the effect is strongly sex specific via unknown mechanisms. Meyer *et al*. found that prenatal cocaine in rats led to a loss of cardioprotection associated with preconditioning as well as reduced PKCε mRNA in the hearts of male offspring while females maintained cardioprotection and normal PKCε levels in the face of prenatal exposure [97]. Interestingly, prenatal METH exposure has been shown to significantly increased cardiac injury in female but not male hearts subjected to ischemia and reperfusion via the Langford isolated heart system; PKCε expression was significantly downregulated in female offspring of cocaine-exposed mothers but not in male offspring in the associated study [98]. METH and cocaine exert their effects via indirect stimulation of dopaminergic and adrenergic receptors despite different specific mechanisms of action, so the stark reversal of their sex-specific effects in combination with consistent molecular findings is intriguing. Overall, these findings suggest that early exposure to psychostimulants may lower the threshold for induction of cardiovascular disease through DNA methylation-mediated changes in gene expression and negatively impact recovery from ischemic insult, potentially leading to an increased incidence and severity of adverse cardiovascular events in the adult population.

### Aging

The aging process is an inevitable, gradual degeneration of tissue and organ function associated with an increased risk of morbidity and mortality [99]. An increasing number of studies have linked aging to both genetic and epigenetic changes, and the reversible nature of epigenetic marks makes them of particular interest for therapeutics targeting age-related decline and disease [100]. Prenatal exposure to psychostimulants has been found to enhance age-related degeneration in rodents, with a study by Church *et al*. finding that adult offspring of cocaine-exposed females had a 7–12% reduction in lifespan and decreased body weights in old age when compared to unexposed offspring [93, 101]. Importantly, exposed offspring did not significantly differ in body weight or other parameters during adolescence and young adulthood despite initially low body weights during the neonatal period. These findings suggest that epigenetic marks were set in development and persisted until old age, concomitant with diverse negative physiological effects. The effect on lifespan was strongest in female offspring, however, significant differences were observed in both sexes. While direct links between epigenetic mechanisms and aging phenotypes related to perinatal drug exposure are lacking in the literature, numerous epigenetic changes including global loss of histone marks, global hypomethylation with site-specific hypermethylation (i.e. epigenetic drift), and increased transcriptional noise have been found to occur in the aging genome [99, 102–105]. Environmental factors during the perinatal period including nutrition, stress, and lead (Pb) exposure have been shown to shift epigenetic markers of aging [106, 107], however, future animal studies evaluating epigenetic measures of aging, total lifespan, and other markers of age-related decline will be required before strong conclusions regarding perinatal drug exposure’s effects on lifespan can be reached.

### Potential for Second Generation and Transgenerational Effects

Transgenerational epigenetic inheritance can be defined as the process through which an environmental exposure disrupts germline epigenetic marks causing permanent changes in the gene regulatory profile of subsequent generations [108]. For maternal exposures, assessment of true transgenerational effects requires study out to the F3 generation due to the direct exposure of F1 embryo and F2 primordial germ cells (PGCs) to substances consumed by the F0 female during pregnancy. In contrast, paternally transmitted transgenerational effects can be observed in the F2 generation due to ongoing spermatogenesis in F1 adulthood [109]. Much remains unknown about whether illicit drug use during critical stages of development permanently modifies the epigenetic landscape within subsequent, unexposed generations. Importantly, transgenerational persistence of environmentally induced phenotypes is the exception rather than the rule in mammals due to the highly sequestered germline and resetting of DNA methylation marks during early embryogenesis and formation of PGCs. Currently, there are no studies examining the epigenetic or phenotypic transgenerational effects of cannabinoids or METH exposure beyond the F1 generation, however, studies exploring early life parental cocaine and morphine use provide some evidence for discussion. Wimmer *et al*. identified histone modifications and increased cocaine-induced drug seeking in Sprague–Dawley rat F1 males, however, no behavioral changes were observed in male F2 grand-offspring [110]. Similar results were seen in a study by Yaw *et al*., where F2 rat offspring of cocaine-exposed males did not show enhanced cocaine preference when compared to controls [111]. Using the same strain of rats, however, Le *et* *al* found that high incentive to cocaine responding F0 rat behavior was recapitulated in the F2 grand-offspring [112]. These behavior changes were correlated with 1244 differentially methylated CpG sites identified between exposed F0 and unexposed F0 sperm as well as 544 differentially methylated CpG sites in exposed F1 sperm. Studies exploring epigenetic alterations, downstream pathways, and behavior in F2 offspring are needed to validate these findings, but evidence of continued epigenetic dysregulation in the F1 germ cells implies that transgenerational transmission of the phenotype may be possible. Paternal morphine use can also result in intergenerational phenotypes with exposure during adolescence causing changes in neurobehavior in male F1 offspring by altering the rewarding effects of morphine and the spontaneous burst firing of VTA dopaminergic neurons [113]. Deficits in the F2 generation have also been seen with maternal drug exposure, with male grand-offspring of female rats exposed to morphine prior to pregnancy showing a reduced ability to find a hidden platform during the Morris water maze spatial learning test [114]. Hippocampal expression of the repressive transcription factor Mecp2 and the repressive histone deacetylase Hdac2 were also found to be increased in generationally exposed F2 males compared to F2 progeny of non-morphine consuming mothers. These results indicate that morphine consumption, even prior to pregnancy, is associated with deficits in spatial memory correlated with differential expression of epigenetic regulators in the brain.

Little is known regarding the effects of generational exposure to other plant-derived drugs and phytochemicals on subsequent offspring health. The ubiquitous, licit stimulant caffeine has been shown to alter neuroendocrine metabolic programing in F2 rat progeny [115]. F2 offspring exhibited altered neuroendocrine metabolic states and greater levels of corticosterone after chronic stress, along with impaired glucose and lipid metabolism. Other plant-based phytochemicals such as flavonoids, stilbenes, and lignans induce physiological and epigenetic changes primarily seen within F1 individuals [116]. While the generational effects of other plant-based compounds strengthen the argument that illicit drugs, many of which are derived from botanicals, may have transgenerational effects, studies exploring epigenetic inheritance beyond the F2 generation are needed to understand whether parental substance use persistently alters the offspring epigenome.

## Conclusion

It is evident based on the recent literature that illicit drugs encountered *in utero* are effectors of the epigenome, with impacts on DNA methylation, histone modification, and expression of small RNAs present in pathways as diverse as reward, memory, aging, and cardiovascular disease. The number of drugs and phenotypes with measured epigenetic effects is relatively few thus far, however, and further investigation of these effects will be vital for a complete understanding of the consequences of developmental exposure to illicit drugs and other toxicants. Direct linkage of epigenetic changes to tissue-specific mRNA and/or protein levels for candidate gene networks is also vital to identify specific causative or contributory epigenetic changes associated with a given phenotype. Exposure of the developing fetus represents a perturbation of a key epigenetic critical period during which exogenous chemicals have the potential to adversely affect not only the F1 but also the F2 generation, which can be exposed during F1 PGC development. Not even FDA-approved pharmaceuticals are routinely tested for adverse germline effects, and illicit drugs taken at unknown doses and purities are of great concern for the eventual adult health of children and grand-children being exposed *in utero* [117]. Part of the solution to this problem will involve improving the clinical applicability of animal model exposures: cannabis, for example, is composed of hundreds of phytocannabinoid compounds and terpenes that are thought to modulate one another’s activity at different concentrations (i.e. the ‘entourage effect’), and experimental prenatal exposure to THC or its synthetic analogs alone may not capture the true effects of maternal cannabis use during pregnancy. Likewise, experimental drug exposure windows should reflect the pattern of human use whenever possible; doing so may help to resolve instances of opposite or contradicting effects found with the wide variety of short-term exposure paradigms in the literature. Marked and consistent sex-specific effects are also clear in the studies assembled, and further interrogation of this phenomenon will be vital for well-informed treatment of associated adult-onset diseases. Lastly, the influence of altered maternal care of offspring can itself impact the epigenetic legacy. The effects of serial programing of maternal care (i.e. behavior recapitulated each generation) versus the direct impact of drug use on the inherited epigenome can be difficult to distinguish. For example, increased maternal care has been negatively correlated with cocaine and alcohol use in rats, which in turn impact the epigenome of offspring [118]. These effects may also significantly obfuscate direct intergenerational drug effects on the methylome in animal models.

Given the relatively small body of existing literature, many high-impact avenues of investigation in the field warrant attention. In combination with addressing the challenges and potential confounds identified above, broad future directions for the field may include epigenetic mechanisms of multi-drug phenotype interactions (e.g. cannabis and opioid use [119, 120]), within-drug interactions (e.g. cannabinoid ratios), and expansion of targeted editing strategies for epigenetic candidate loci. Ultimately, much work remains to be done in characterizing the epigenetic consequences of developmental exposure to illicit substances on the health of the resulting adult; connecting the molecular signatures of historical exposure to mechanisms of adult disease will be imperative in providing robust preventative and therapeutic measures moving forward.
